# Adaptive Multi-Scale Fusion Enhanced RT-DETR for Efficient Cyanobacteria Detection in Microscopic Images

**DOI:** 10.3390/biology15120970

**Published:** 2026-06-20

**Authors:** Jianxing Li, Shizhi Zheng, Yu Chen, Kan Luo

**Affiliations:** 1 School of Electrical and Information Engineering, Fujian University of Technology, Fuzhou 350118, China; lijx@fjut.edu.cn (J.L.); 2241905011@smail.fjut.edu.cn (S.Z.); 2 Fuzhou Industrial Integration Automation Technology Innovation Center, Fuzhou 350118, China; 3 State Key Laboratory of Digital Medical Engineering, School of Instrument Science and Engineering, Southeast University, Nanjing 210096, China

**Keywords:** cyanobacteria detection, microscopic image analysis, RT-DETR, multi-scale feature fusion, Wise-IoU, object detection

## Abstract

Accurate and efficient detection of cyanobacteria in microscopic images is important for automated water-quality monitoring, but remains challenging because of complex aquatic backgrounds, large scale variation, and uneven sample quality. This study proposes an adaptive multi-scale fusion enhanced RT-DETR framework for cyanobacteria detection. The baseline RT-DETR-R18 is improved by incorporating the SeFaster module for efficient feature extraction, the high-level screening-feature fusion pyramid network for semantic-guided multi-scale fusion, and the Wise-IoU loss for more stable localization learning under mixed-quality samples. Experiments on the reorganized EMDS-7 dataset show that the proposed method achieved 79.05% mAP@0.5, 66.03% mAP@0.5:0.95, 16.31 M parameters, 54.6 G FLOPs, and 70.85 FPS. The proposed model also obtained the highest mAP@0.5 across the seven cyanobacteria categories. Moreover, cross-dataset evaluations further suggest the stability and transferability of the model. These results indicate that the proposed framework demonstrates potential for effective cyanobacteria detection in microscopic images with a good balance between detection accuracy and computational efficiency.

## Simple Summary

Cyanobacteria, often called blue-green algae, can harm water quality and may threaten human, animal, and environmental health. Detecting them under a microscope is important, but manual identification is slow and difficult because images often contain complex backgrounds, organisms of different sizes, and samples of uneven quality. This study aimed to develop a faster and more accurate computer-based method to find cyanobacteria in microscopic images. We improved an existing image-detection model by helping it extract useful visual features more efficiently, combine information from different image scales, and learn object locations more reliably when image quality varies. Tests on a reorganized public dataset containing seven types of cyanobacteria showed that the proposed method detected cyanobacteria accurately while keeping the model relatively small and fast. It achieved the best average detection performance across the seven categories and could process images quickly enough for practical use. These findings suggest that the proposed method shows potential to support automated water-quality monitoring, reduce the workload of experts, and help detect potential water pollution risks more efficiently.

## 1. Introduction

Cyanobacterial blooms have become an important issue in freshwater environments. Driven by climate change and nutrient enrichment, their occurrence has increased in many lakes and reservoirs worldwide [1,2]. Excessive cyanobacterial growth can reduce water transparency, alter dissolved oxygen conditions, and disrupt the ecological balance of aquatic systems [3,4]. In addition, several cyanobacterial genera produce toxins that threaten aquatic ecosystems, drinking water safety, and human health. For example, *Microcystis*, a globally prevalent bloom-forming genus, is well known for producing microcystins. Certain *Raphidiopsis* species are capable of producing cylindrospermopsin and other harmful metabolites, while *Anabaenopsis* has also been associated with harmful cyanobacterial blooms in freshwater environments [5,6,7,8]. Therefore, accurate and timely monitoring of cyanobacteria is important for water quality assessment and the early warning of bloom-related risks.

Current cyanobacteria monitoring primarily relies on analytical, molecular, and microscopic methods [9,10]. Among these, microscopic examination serves as the most prevalent approach for the identification of algal species [11]. However, traditional manual microscopy inherently consumes significant time, requires extensive human effort, and depends heavily on the expertise of the observer, making it impractical for rapid and extensive screening [10,12]. To overcome these critical bottlenecks, automated analysis of microscopic images has emerged as a crucial solution. Early automated algal analysis mainly relied on conventional image processing and hand-crafted features [13,14]. Although these methods can be effective under controlled conditions, their performance is often sensitive to background complexity, contrast variations, overlapping objects, and morphological diversity, which limits their robustness and generalizability. With the rapid development of computer vision, deep learning-based detection methods have shown clear advantages in microscopic image analysis [15]. By learning discriminative representations directly from image data, these methods can improve both detection accuracy and automation efficiency. Recent studies have demonstrated the feasibility of deep learning for algal or microalgal detection tasks [16,17,18,19,20,21]. These studies suggest that effective feature extraction, multi-scale representation, and efficient model design are all important for improving detection performance in microscopic images.

Despite this progress, efficient cyanobacteria detection in microscopic images still has room for improvement. Existing studies are still dominated by broader microalgae and algae detection tasks, while research specifically focused on cyanobacteria remains relatively limited. More importantly, cyanobacteria present several task-specific challenges, including substantial morphological variations from unicellular to filamentous forms, pronounced scale differences, visual similarity among related taxa, and class imbalance [22]. These characteristics place higher demands on multi-scale representation, fine-grained discrimination, and computational efficiency. Therefore, improving deep learning models according to the visual characteristics of cyanobacterial microscopic images remains necessary for achieving accurate and efficient detection.

In this study, we propose an enhanced Real-Time Detection Transformer (RT-DETR) framework adapted for cyanobacteria detection in microscopic images, aiming to address key challenges such as redundant aquatic backgrounds, multi-scale variations, and overall morphological similarities among targets. To better adapt to the unique characteristics of cyanobacterial images, we introduce three key innovations. First, a squeeze and excitation faster feature extraction (SeFaster) module is introduced into the backbone for more efficient feature extraction. Second, a high-level screening-feature fusion pyramid network (HSFPN) [23] is incorporated to enhance multi-scale feature fusion. Third, the Wise-IoU (WIoU) [24] loss is adopted to reduce the influence of uneven sample quality during training. Finally, experiments conducted on the public Environmental Microorganism Image Dataset Seventh Version (EMDS-7) [25] demonstrate the effectiveness of the proposed method.

## 2. Related Work

With the development of deep learning, convolutional neural network (CNN)-based detectors have become the main approach for automated microalgae detection. Most existing studies are built on one-stage frameworks, especially the YOLO series [26] and SSD [27]. Current improvements mainly follow two lines. One line focuses on improving detection accuracy in complex microscopic images. Typical strategies include the use of attention mechanisms, enhanced feature pyramid structures, optimized neck and head designs, and improved localization loss functions to better handle small targets, dense distributions, and large-scale variations. For example, Liang et al. [16] incorporated BiFPN and CBAM into EfficientDet and reported improved performance. Duan et al. [17] optimized the neck and detection head of YOLOv5s, while Chu et al. [18] introduced SPP and GIoU loss into YOLOv3 to improve detection under overlapping conditions. Further refinements have also been explored in YOLOv7-based models. Ding et al. [19] introduced a CAGS module and SIoU loss in FE-YOLO, and Liu et al. [20] proposed Microalgae-YOLO with a patch training-overall prediction framework, a parallel feature extraction network, and TIoU loss. These studies show that stronger feature extraction and more effective multi-scale fusion are important for improving algae detection in microscopic images.

Another line of research focuses on improving computational efficiency for practical deployment. In this direction, lightweight backbones and efficient modules are introduced to reduce parameters and floating-point operations while preserving competitive performance. Liu et al. [21] proposed Algae-YOLO by combining ShuffleNetV2 and GhostBottleneck modules with YOLOv5s, which greatly reduced model size while maintaining comparable detection performance. These studies indicate that lightweight design is important for real-time monitoring tasks, especially when large numbers of samples must be processed under limited hardware conditions.

Although CNN-based methods provide a useful foundation for cyanobacteria detection, they inherently excel at extracting local visual patterns, such as textures and edges. However, in cyanobacterial microscopic images, targets frequently exhibit severe spatial overlap and complex morphological variations, ranging from isolated cells to elongated filamentous aggregates. Interpreting these structures depends not only on localized features but also heavily relies on their broader spatial arrangement and global contextual information. Consequently, relying exclusively on local receptive fields can lead to insufficient modeling of structural relationships and spatial dependencies among dense targets. For this reason, Transformer-based architecture provides a meaningful direction for further study, as their intrinsic global context modeling is fundamentally better suited for capturing these overarching spatial relationships.

**Figure 1:**
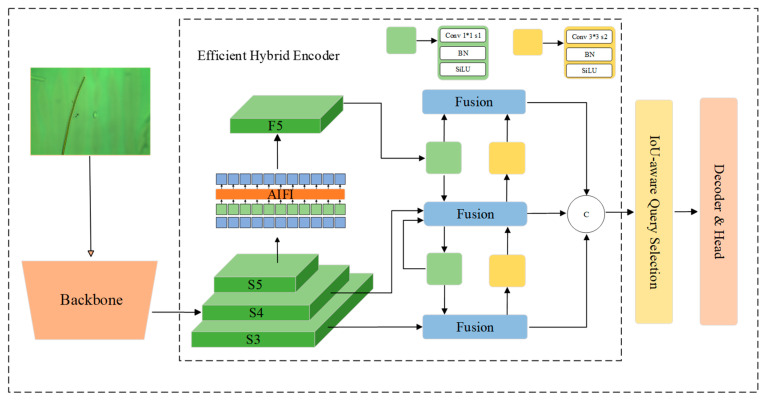
Overall architecture of the baseline RT-DETR-R18 model.

Models such as DETR, Deformable DETR, and RT-DETR introduce self-attention into object detection [28,29,30]. Among them, RT-DETR is particularly relevant because it combines the global modeling capability of the Transformer with real-time detection efficiency. As shown in Figure 1, the basic RT-DETR-R18 mainly consists of a backbone, an Efficient Hybrid Encoder, an IoU-aware query selection module, and a Transformer decoder with detection heads. The backbone typically employing ResNet [31] or HGNet [30], extracts multi-scale features from the input image, while the Efficient Hybrid Encoder, composed of the Attention-based Intra-scale Feature Interaction (AIFI) module and the CNN-based Cross-scale Feature Fusion (CCFM) module, strengthens semantic interaction and cross-scale fusion with relatively low computational cost. The selected informative features are then used as object queries and refined by the decoder to produce the final classification and localization results. This design makes RT-DETR a suitable baseline for cyanobacteria detection.

RT-DETR has shown strong performance in general object detection [30]. However, considering the aforementioned complex characteristics of cyanobacterial microscopic images, its direct application still requires further adaptation. Therefore, this study proposes an enhanced RT-DETR framework featuring three targeted innovations.

## 3. Adaptive Multi-Scale Fusion Enhanced RT-DETR

**Figure 2:**
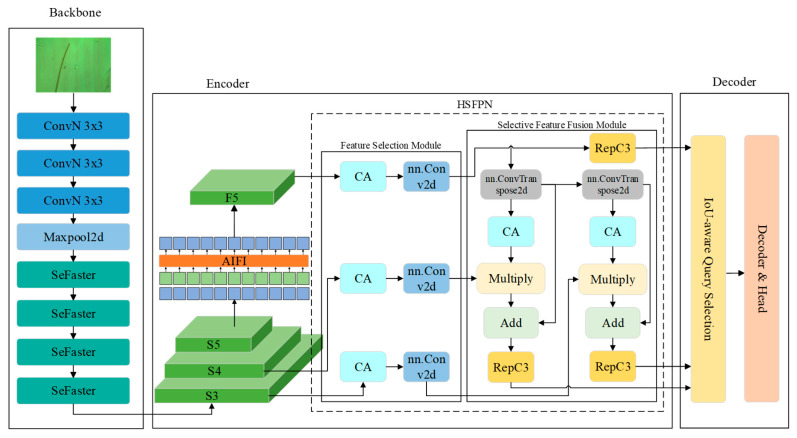
Overall architecture of the adaptive multi-scale fusion enhanced RT-DETR.

The overall architecture of the proposed adaptive multi-scale fusion enhanced RT-DETR is shown in Figure 2. The model is developed from the basic RT-DETR-R18 and retains its end-to-end detection pipeline, including backbone feature extraction, efficient hybrid encoding, IoU-aware query selection, and Transformer decoding.

The specific processing flow of the model proceeds as follows. The input image is first processed by several convolutional layers and four SeFaster blocks to generate hierarchical feature maps. Three backbone outputs, denoted as S3, S4, and S5, are retained for subsequent multi-scale representation. Among them, S3 contains richer spatial details, S4 provides intermediate structural information, and S5 carries stronger semantic information. The deepest feature, S5, is processed by the AIFI module to strengthen intrascale semantic interaction, yielding the refined feature F5. Subsequently, the multilevel features S3, S4, and F5 are integrated by an HSFPN-based fusion module adapted to the RT-DETR encoder, where high level semantics guide the screening and integration of lower level details. The fused features are then fed into the IoU-aware query selection module to generate informative object queries, which are subsequently refined by the Transformer decoder and detection head for final prediction. During the training, the WIoU loss is adopted to dynamically adjust regression gains, optimizing the bounding box regression process under varying sample quality. The following subsections describe the design of SeFaster, HSFPN, and WIoU, respectively.

### 3.1. Squeeze-and-Excitation Faster Feature Extraction

**Figure 3:**
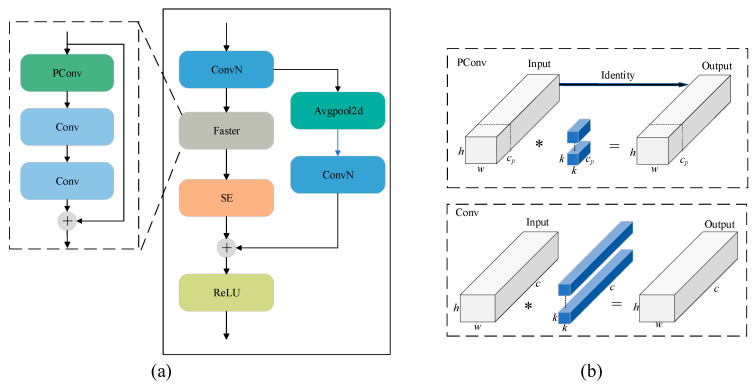
Detailed structure of the proposed SeFaster module. (**a**) The overall architecture of the module; (**b**) Comparison of convolution operations within the module.

The detailed structure of the proposed SeFaster module is shown in Figure 3a. This module is designed to improve feature extraction efficiency specifically for cyanobacterial microscopic images. In such images, the aquatic background often occupies a large portion of the field of view, so the extracted feature maps may contain substantial redundant information. Conventional convolutional layers, such as those utilized in the baseline backbone, process all spatial regions and channels equally. This lack of selective focus not only increases computational cost but can also weaken the representation of subtle cyanobacterial structures. To address these challenges, we considered optimizing the feature extraction process from both spatial and channel perspectives. Partial Convolution (PConv) [32] offers an efficient approach to reducing spatial redundancy and computational overhead by operating only on a subset of channels. Concurrently, the Squeeze-and-Excitation (SE) mechanism [33] is highly effective at modeling channel-wise dependencies to adaptively emphasize informative features. Drawing on these complementary advantages, we combined them to construct the SeFaster module. This synergistic design enables the backbone to effectively filter out redundant background computation while preserving a necessary degree of sensitivity to crucial cellular features.

As shown in Figure 3, SeFaster is organized as a residual feature extraction unit. The input feature first passes through a normalization and convolution stage and is then processed by the Faster block for efficient spatial feature extraction. The output is subsequently refined by the SE module for channel-wise recalibration. In parallel, a shortcut branch preserves the original feature information. The outputs of the main branch and the shortcut branch are then fused by element-wise addition, followed by a ReLU activation. This design retains the advantages of residual learning while improving both computational efficiency and feature selectivity. Within the module, the Faster block mainly supports efficient spatial modeling, whereas the SE branch enhances channels that are more relevant to cyanobacterial morphology and fine structural details.

The core of SeFaster is the Faster block, in which PConv is used to replace full convolution for part of the spatial processing. In standard convolution, the kernel operates on all input channels, which leads to relatively high floating-point operations (FLOPs) and memory access cost (MAC). By contrast, PConv performs convolution only on a subset of channels and leaves the remaining channels unchanged, thereby reducing both computation and memory access. As illustrated in Figure 3b, the computational cost and memory access of standard convolution, which are $h\times w\times k^{2}\times c^{2}$ and h×w×2c reduce to $h\times w\times k^{2}\times c_{p}^{2}$ and $h\times w\times 2c_{p}$, respectively [32]. Under the typical setting of $c_{p}/c$ = 1/4, the FLOPs and MAC of PConv are reduced to only 1/16 and 1/4 of those of conventional convolution, respectively. This design contributes to feature extraction efficiency while retaining the spatial information required for subsequent detection.

While the Faster block contributes to reducing computational redundancy, distinguishing subtle cyanobacterial structures from complex aquatic backgrounds necessitates further discrimination at the channel level. The SE module is further introduced to model channel-wise dependencies. Specifically, global average pooling is first applied to compress each channel into a compact descriptor, and channel weights are then generated through the excitation operation. These weights are used to recalibrate the feature responses, so that channels containing more informative cyanobacterial features receive greater emphasis. In this way, SeFaster combines the efficiency advantage of PConv with the channel selection capability of SE, thereby improving both computational efficiency and feature discrimination.

### 3.2. High-Level Screening-Feature Fusion Pyramid Network

**Figure 4:**
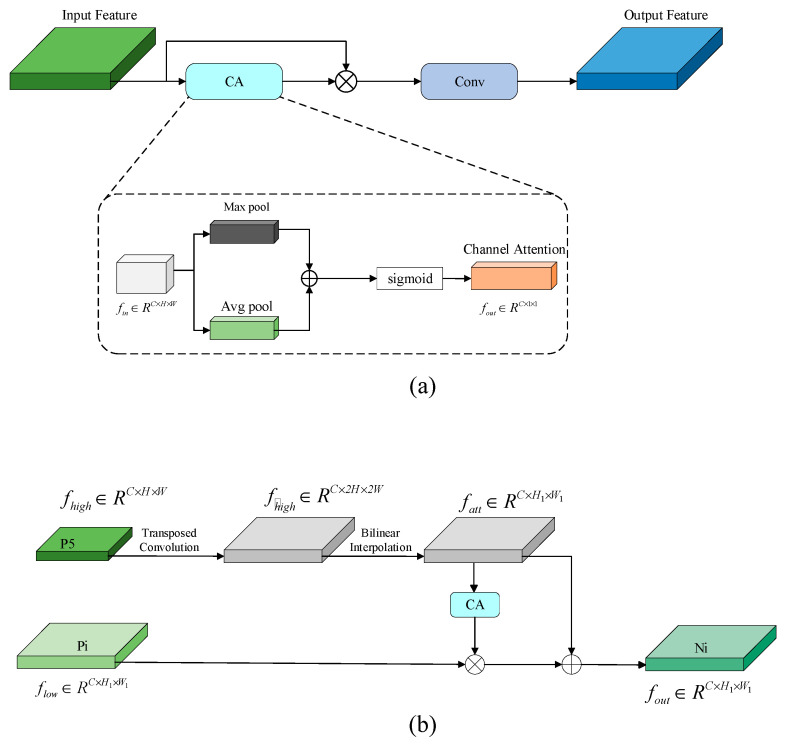
Detailed structures of HSFPN modules: (**a**) Feature Selection module (FSM); (**b**) Selective Feature Fusion (SFF) module.

In cyanobacterial microscopic images, targets frequently exhibit substantial scale variations, appearing as small individual cells, compact colonies, or elongated filamentous aggregates within the same field of view. Under such complex conditions, the simple cross-scale feature aggregation performed by the original CCFM in the baseline can be insufficient. This is primarily because low-level features contain rich spatial details but also carry more background interference, whereas high-level features provide stronger semantics but tend to lose fine structural information. To address these challenges and mitigate the limitations of simple aggregation to a certain extent, the original CCFM is replaced by incorporating the HSFPN. This framework introduces a high-level semantic guidance mechanism designed to screen and fuse multi-scale features more adaptively. As illustrated in Figure 4, HSFPN consists of two components: the Feature Selection Module (FSM) and the Selective Feature Fusion (SFF) module. The former is utilized to recalibrate feature responses prior to fusion, while the latter performs semantic-guided integration between high-level and low-level features. Together, these two modules aim to preserve discriminative details while contributing to the reduction of redundant information during cross-scale feature interaction.

#### 3.2.1. Feature Selection Module

The structure of the FSM is shown in Figure 4a. This module is designed to enhance informative channels before feature fusion. Given an input feature map, FSM first applies a Channel Attention (CA) operation to estimate the importance of each channel. As illustrated in Figure 4, the input feature is processed by parallel max pooling and average pooling branches to aggregate complementary channel descriptors. The pooled responses are then combined and passed through a sigmoid activation to generate channel-wise attention weights. These weights are multiplied with the original feature map so that channels containing more useful information receive larger responses, while less informative channels are suppressed. A convolution layer is then applied to obtain the refined output feature.

The role of FSM is to improve the quality of features before cross-scale fusion. In cyanobacterial microscopic images, low-level features often preserve boundary and texture information, but they may also contain irrelevant background responses. By introducing channel-wise selection, FSM helps the network focus more on biologically relevant patterns, such as cyanobacterial contours, local morphology, and fine structural details. This makes the subsequent fusion process more selective and reduces the influence of redundant or noisy responses.

#### 3.2.2. Selective Feature Fusion Module

After feature selection, multi-scale features are integrated by the SFF module, as shown in Figure 4b. Unlike direct additive fusion, SFF follows a semantic-guided fusion strategy. Specifically, the high-level feature $f_{high}\in R^{C\times H\times W}$ is first up-sampled by transposed convolution to generate $f_{\hat{high}}\in R^{C\times 2H\times 2W}$, and then adjusted by bilinear interpolation to yield $f_{att}\in R^{C\times H_{1}\times W_{1}}$.

(1) $$f_{att}=BL(TConv(f_{high}))$$

where the TConv() and BL() are the operators of transposed convolution and bilinear interpolation, respectively. And then the aligned high-level feature $f_{att}$ is passed through a CA module, and the resulting attention response is multiplied with the low-level feature $f_{low}\in R^{C\times H_{1}\times W_{1}}$. The filtered low-level feature is then added to the aligned high-level feature to produce the fused output $f_{out}\in R^{C\times H_{1}\times W_{1}}$. The overall process of feature selection and fusion is formulated in Equation (2).

(2) $$f_{out}=f_{low}\times CA(f_{att})+f_{att}$$

In this way, the high-level semantic information acts as a filter that selects informative spatial responses from the low-level feature map. SFF preserves the detailed localization information of low-level features while using high-level semantics to suppress irrelevant responses and strengthen task-relevant structures. Compared with the original CCFM, HSFPN provides a more selective fusion mechanism for cyanobacterial detection. FSM first improves feature quality through channel-wise screening, and SFF then performs semantic-guided integration across scales. This design is better suited to cyanobacterial microscopic images, where targets vary greatly in size and often appear in dense, overlapping, or morphologically complex forms.

### 3.3. Wise-IoU Loss Function

Cyanobacterial microscopic images often contain blurred targets, overlapping instances, partial occlusion, and substantial morphological variation. As a result, the training data exhibit uneven sample quality, which may affect the stability of bounding box regression. The baseline RT-DETR employs GIoU as the regression loss and treats all samples with a fixed optimization emphasis. Under mixed-quality conditions, this strategy may bias regression learning toward easier and higher-quality examples.

To better exploit such mixed-quality datasets, a more adaptive regression strategy is required; therefore, WIoU is adopted in this study. Unlike GIoU, WIoU dynamically adjusts the regression gain according to prediction quality. By mitigating the disproportionate influence of extreme samples and assigning greater emphasis to informative ordinary samples, WIoU facilitates more efficient learning from uneven datasets, thereby contributing to the overall stability of localization. The formulation of the adopted WIoU loss is detailed as follows.

The IoU loss is first defined in Equation (3),

(3) $$L_{IoU}=1-IoU$$

where IoU denotes the intersection over union between the predicted box and the ground-truth box. Based on this term, the adopted WIoU loss is formulated in Equation (4),

(4) $$L_{WIoU}=rR_{WIoU}L_{IoU}$$

where r denotes the dynamic non-monotonic gain factor [24] and $R_{WIoU}$ denotes the distance attention term defined in Equation (5).

(5) $$R_{WIoU}=\exp(\frac{{\left(x-x_{gt}\right)}^{2}+{\left(y-y_{gt}\right)}^{2}}{{\left(W_{g}^{2}+H_{g}^{2}\right)}^{*}})$$

In Equation (5), (x,y) and $\left(x_{gt},y_{gt}\right)$ are the center coordinates of the predicted box and the ground-truth box, respectively, while $W_{g}$ and $H_{g}$ represent the width and height of the minimum enclosing box covering both boxes.

## 4. Experiments and Results

### 4.1. Dataset Description

This study uses the EMDS-7 [25], a publicly available microscopic image dataset for object detection. According to the dataset release and the corresponding paper, EMDS-7 contains 2365 images and 13,216 labeled objects collected from lakes and rivers in Shenyang, China, under a 400× optical microscope. The dataset is provided with XML-format annotations and is intended for environmental microorganism detection research. To match the objective of this study, the original labels were reorganized into nine categories. Seven cyanobacterial genera, namely *Oscillatoria*, *Phormidium*, *Spirulina*, *Microcystis*, *Coelosphaerium*, *Anabaenopsis*, and *Raphidiopsis*, were retained as the primary detection targets. The remaining non-target classes were merged into two broader categories, Background Algae and Zooplankton, so that the model could better learn cyanobacterial targets under realistic aquatic interference. This reorganization reduces label fragmentation among non-target microorganisms while preserving the categories most relevant to cyanobacteria detection. The resulting class distribution is summarized in Table 1.

### 4.2. Experimental Setting and Evaluation Metrics

The processed dataset was divided into training, validation, and test sets at a ratio of 6:2:2 using a fixed random seed (Seed: 903). To mitigate the evaluation variance associated with severe class imbalance, an image-level stratified splitting strategy was employed. This approach helps ensure that minority categories are proportionally represented in the test set. The detailed distribution of images and instances across all splits is provided in Table S2 in the Supplementary Materials. To ensure a rigorous evaluation and prevent data leakage, the dataset partitioning was performed prior to data augmentation. Data augmentation was applied exclusively to the training set, ensuring that augmented variants of a single original image did not appear across different splits. To increase the diversity of the training data, the training set was expanded by a factor of five, generating four augmented images for each original training image. The offline augmentation pipeline consisted of horizontal flipping with a probability of 50%, random rotation selected from 0°, 90°, 180°, and 270°, and brightness and contrast adjustments with factors uniformly sampled between 0.8 and 1.2.

All experiments were conducted on a 64-bit Windows 10 operating system. The hardware platform consisted of an Intel i5-7400 CPU and an NVIDIA RTX 3060 GPU with 12 GB of memory. The software environment was based on Python 3.11 and PyTorch 2.4.0, with CUDA 12.4 used for hardware acceleration. To ensure fair baseline comparisons, all models were trained from scratch without using pre-trained weights and were trained using the same dataset partition, input resolution, optimizer settings, batch size, and number of training epochs. Specifically, input images were resized to 640 × 640 pixels, and all models were optimized using the AdamW optimizer with an initial learning rate of 0.0001 and a batch size of 16 for 150 epochs. During evaluation, a unified confidence threshold of 0.001 was applied. For YOLO-family baselines, the Non-Maximum Suppression (NMS) IoU threshold was set to 0.7, whereas Transformer-based architectures (e.g., RT-DETR and D-FINE) inherently bypassed NMS. For FPS measurement, all trained models were exported to TensorRT format and evaluated on the RTX 3060 GPU with a batch size of 1. The final speed was calculated by averaging the inference time over 500 evaluation iterations following a 100-frame warm-up period.

To evaluate the proposed method, standard object detection metrics were used from three aspects: detection accuracy, model complexity, and inference efficiency. Detection accuracy was assessed by Precision (P), Recall (R), and mean Average Precision (mAP). Precision measures the proportion of correctly predicted positive samples among all predicted positives, whereas Recall measures the proportion of actual positive samples that are correctly detected. The mAP is calculated as the mean of the Average Precision (AP) over all classes. These metrics are mathematically formulated as shown in Equations (6)–(8).

(6) $$P=\frac{TP}{TP+FP}$$

(7) $$R=\frac{TP}{TP+FN}$$

(8) $$mAP=\frac{1}{N}\sum_{i=1}^{N}AP_{i}$$

where *TP*, *FP*, and *FN* denote the numbers of true positives, false positives, and false negatives, respectively, and $AP_{i}$ denotes the average precision of the *i-th* class. In addition, model complexity and inference efficiency were evaluated by Parameters, FLOPs, and FPS. Parameters denote the total number of trainable variables, FLOPs measure the computational cost of a single forward pass, and FPS reflects the inference speed of the model.

### 4.3. Results of Comparative Experiments

To evaluate the proposed method, comparative experiments were conducted against representative YOLO-family detectors and several non-YOLO real-time detectors under the same dataset split and experimental settings. The YOLO baselines [34,35,36,37,38,39] included both small and medium variants, covering different accuracy-efficiency trade-offs. The non-YOLO baselines included RT-DETR-R18 [30], D-FINE-S [40], and RF-DETR-S [41].

The overall results are reported in Table 2. Among the YOLO-family baselines, YOLOv11m achieved the strongest overall performance, with 77.74% mAP@0.5 and 65.14% mAP@0.5:0.95. The small models, such as YOLOv12s, YOLOv13s, and YOLO26s, ran faster and used fewer parameters, but their detection accuracy was generally lower. This result indicates that increasing model capacity remains helpful for this task, but the gain in accuracy is often accompanied by higher computational cost. Among the non-YOLO baselines, RF-DETR-S achieved the highest mAP@0.5 and mAP@0.5:0.95, while D-FINE-S showed relatively low precision and recall on this dataset. Compared with RT-DETR-R18, the proposed method improved mAP@0.5 from 75.85% to 79.05% and mAP@0.5:0.95 from 61.20% to 66.03%. At the same time, the parameter count decreased from 20.10 M to 16.31 M, FLOPs decreased from 58.3 G to 54.6 G, and FPS increased from 66.27 to 70.85. These results show that the proposed modifications improve both detection accuracy and efficiency over the baseline RT-DETR.

Compared with all baseline models, the proposed method achieved the best overall mAP@0.5 and mAP@0.5:0.95, while maintaining competitive inference speed. Although its precision (83.70%) was slightly lower than that of YOLOv11m (83.92%), it obtained the highest recall (73.67%) and the best comprehensive detection performance. Relative to YOLOv11m, the proposed method improved mAP@0.5 by 1.31 percentage points and mAP@0.5:0.95 by 0.89 percentage points, while reducing parameters by 3.75 M, reducing FLOPs by 13.6 G, and increasing FPS by 6.87. This suggests that the proposed model provides a better balance between detection accuracy and computational efficiency. To further validate the statistical stability of these reported gains, we estimated the 95% Confidence Intervals (CI) for the primary metrics using a Bootstrap resampling method. Specifically, 1000 iterations of random sampling with replacement were performed on the test set predictions. The resulting CI for the proposed model (95% CI: [72.90%, 83.77%] for mAP@0.5 and [59.99%, 70.95%] for mAP@0.5:0.95) indicate that the performance improvements over the baselines are relatively stable.

The per-class AP@0.5 results are presented in Table 3. To summarize performance on the target task, the mAP@0.5 over the seven cyanobacteria categories is also reported. The proposed method achieved the highest mean value, reaching 75.31%, compared with 73.80% for YOLOv11m and 72.71% for RF-DETR-S. This indicates that the proposed model provides the best overall performance across the seven cyanobacteria categories. Among the target categories, the improvement was most evident for *Oscillatoria*, where the proposed method achieved 71.58%, clearly higher than the other compared models. The proposed method also obtained the best result on *Microcystis*, which has a relatively larger number of samples and therefore provides a more stable basis for comparison. On *Phormidium*, the proposed method remained competitive, although the highest value was achieved by YOLOv8m. For *Spirulina*, the proposed method performed well but did not exceed RT-DETR-R18. For *Raphidiopsis* and *Anabaenopsis*, the proposed method ranked in the middle. A likely reason is that these categories contain relatively few samples, making mAP@0.5 more sensitive to sample fluctuation and less discriminative for model comparison. For the two interference categories, the proposed method also showed strong performance. It achieved the highest AP@0.5 on BA, reaching 87.35%, which suggests better discrimination between cyanobacterial targets and algae-dominated background regions. On Zoo, the proposed method achieved 96.89%, slightly lower than YOLOv8m, but still at a high level overall. These results indicate that the proposed model not only improves detection of the target cyanobacteria categories but also maintains strong recognition ability for major interference categories in complex aquatic microscopic images.

Overall, the experimental results in Table 3 show that the proposed method achieves the best mean performance across the seven cyanobacteria categories while maintaining competitive results on BA and Zoo. This suggests that the model provides a better overall balance between target detection and interference discrimination on the reorganized EMDS-7 dataset.

### 4.4. Results of Ablation Studies

To evaluate the contribution of each proposed component, ablation experiments were conducted under the same experimental settings. Starting from the baseline RT-DETR-R18, data augmentation, SeFaster, HSFPN, and WIoU were introduced individually and in combination. The quantitative results are summarized in Table 4.

As shown in Table 4, data augmentation itself has a substantial impact on model performance. Without data augmentation, the baseline model achieved 59.39% Precision, 64.58% Recall, 61.38% mAP@0.5, and 50.78% mAP@0.5:0.95. After data augmentation was introduced, these values increased to 81.06%, 70.00%, 75.85%, and 61.20%, respectively, while the model complexity remained unchanged. Although data augmentation did not eliminate class imbalance, it substantially improved the overall detection performance and provided a stronger baseline for subsequent module evaluation. Based on the augmented baseline, all three proposed components further improved performance to different extents. When SeFaster was introduced alone, the model achieved gains in both accuracy and efficiency. The mAP@0.5 increased from 75.85% to 76.91%, and mAP@0.5:0.95 increased from 61.20% to 62.48%. At the same time, FLOPs decreased from 58.3 G to 50.9 G, and Params decreased from 20.10 M to 17.10 M. This suggests that SeFaster improves feature extraction while also reducing computational cost. When HSFPN was added alone, the model also showed consistent improvement, especially in localization-related performance. The mAP@0.5 increased to 76.71%, and mAP@0.5:0.95 increased to 63.91%, which is higher than the result obtained by SeFaster alone on this metric. This indicates that the redesigned fusion module is effective for improving cross-scale feature interaction and is particularly helpful for multi-scale cyanobacterial targets. At the same time, HSFPN slightly increased FLOPs, suggesting that its gain comes with additional fusion cost. Using WIoU alone mainly improved regression-related performance. Compared with the baseline, Precision, Recall, mAP@0.5, and mAP@0.5:0.95 all increased, reaching 83.40%, 72.58%, 76.95%, and 62.67%, respectively. Since WIoU does not change the network structure, FLOPs and Params remained the same as those of the baseline. These results suggest that WIoU improves the stability of localization learning under uneven sample quality, without increasing computational cost. The combination of SeFaster and HSFPN produced further gains. With both modules added, the model achieved 77.73% mAP@0.5 and 64.80% mAP@0.5:0.95, while the parameter count remained at 16.31 M. This result shows that the backbone enhancement and the improved fusion strategy are complementary. SeFaster improves efficient feature extraction, whereas HSFPN strengthens feature integration across scales. Furthermore, to separate independent effects from interaction effects, the remaining two-module combinations were evaluated. When SeFaster and WIoU were combined, the mAP@0.5 increased to 77.61% and mAP@0.5:0.95 to 63.36%. The combination of HSFPN and WIoU also showed consistent improvement, achieving 77.71% mAP@0.5 and 64.13% mAP@0.5:0.95. These results suggest that WIoU is complementary to both SeFaster and HSFPN. The best performance was achieved when data augmentation, SeFaster, HSFPN, and WIoU were used together. The final model reached 83.70% Precision, 73.67% Recall, 79.05% mAP@0.5, and 66.03% mAP@0.5:0.95, outperforming all other settings. Compared with the baseline, the full model improved mAP@0.5 by 3.20 percentage points and mAP@0.5:0.95 by 4.83 percentage points, while also reducing FLOPs from 58.3 G to 54.6 G and Params from 20.10 M to 16.31 M. These results show that the proposed components are effective individually and more effective when combined, leading to simultaneous improvement in detection accuracy and computational efficiency.

### 4.5. Results of Visualization Evaluation

**Figure 5:**
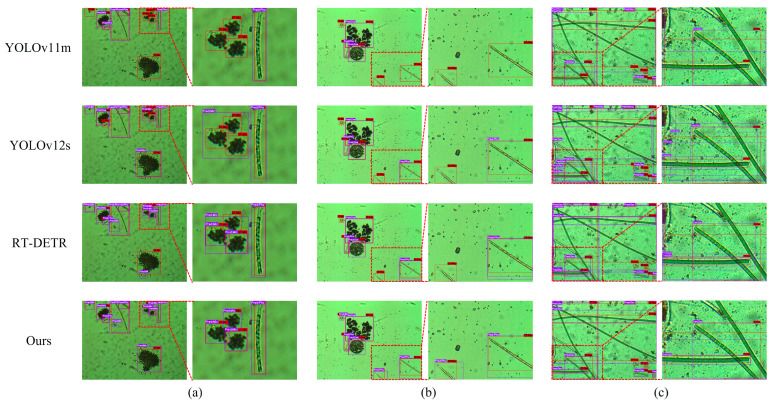
Comparison of detection results on representative EMDS-7 test images. Subfigure (**a**–**c**) correspond to image IDs G003-169, G025-076, and G001-029, respectively. In each subfigure, the ground-truth boxes are shown in red, and the predicted boxes are shown in purple.

To further examine model behavior, qualitative comparisons were conducted on representative EMDS-7 test images containing dense targets, scale variation, and complex aquatic backgrounds. The detection results are shown in Figure 5, where the proposed method is compared with the baseline RT-DETR-R18 and representative YOLO-based detectors.

As shown in Figure 5, the proposed model generally produces more complete detections in challenging cases involving small targets, dense clusters, and filamentous structures. This tendency is more noticeable when targets of different scales appear in the same image or when objects are located near image boundaries. In these cases, the proposed model tends to preserve more valid detections and shows more stable localization on the main target regions. By contrast, the compared models show more incomplete detections or apparent omissions in some crowded or small-target areas. At the same time, the visual differences are not equally obvious in every sample, and some difficult cases remain challenging for all models, especially when the targets are extremely small, weakly contrasted, or heavily overlapped.

**Figure 6:**
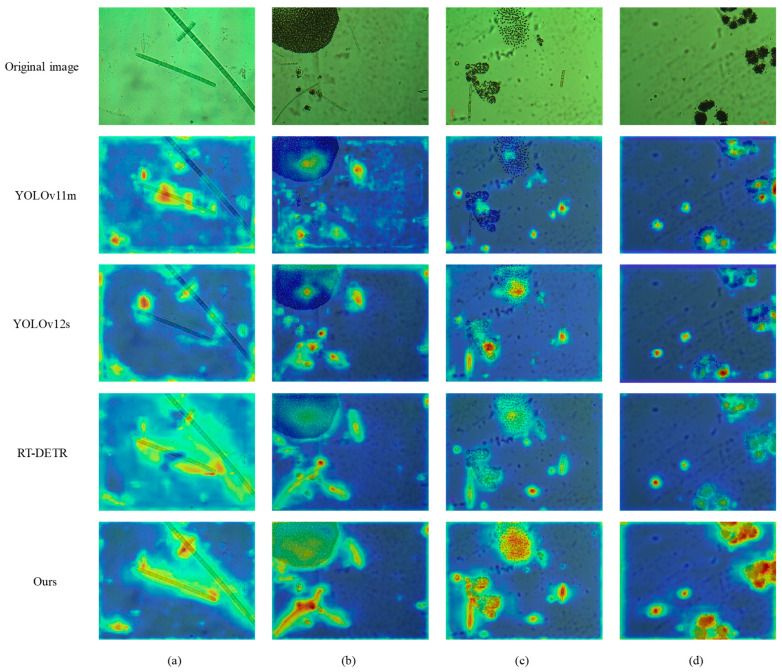
Comparison of Grad-CAM++ heatmaps on representative EMDS-7 test images. Subfigures (**a**–**d**) correspond to image IDs G001-019, G003-072, G003-130, and G003-160, respectively. The first row shows the original images, and the remaining rows show the activation maps of different models. Warmer colors indicate regions with higher model attention, whereas cooler colors indicate regions with lower model attention.

To further compare feature attention, Grad-CAM++ [42] heatmaps were generated for the same set of representative images, as shown in Figure 6. Overall, the proposed model tends to produce activation regions that are more concentrated on target regions and less fragmented across the background. This is more visible in samples containing elongated or aggregated objects, where the activation of the proposed model more often covers the main target area with relatively continuous responses. In contrast, the compared models show more dispersed or locally incomplete activation in several cases, suggesting that their focus on target-related features is less stable under complex backgrounds.

In summary, the visualization results partially enhance the algorithmic interpretability of the model. These heatmaps suggest that the proposed model is better able to focus on target-related visual patterns under cluttered backgrounds and multi-scale conditions. However, these visualizations only illustrate the spatial distribution of algorithmic feature activations and cannot strictly serve as evidence for biological interpretability.

### 4.6. Cross-Dataset Transfer Learning Experiments

To further validate the transferability of the proposed model and alleviate concerns regarding potential evaluation bias from a single dataset split, we conducted cross-dataset transfer learning experiments. We utilized two independent public datasets: the Marine-MicroalgaeDetection [43], which contains 8 classes, and VisAlgae 2023 [44], which consists of 6 classes. For the Marine-MicroalgaeDetection dataset, the images were divided into 430 for training, 107 for validation, and 430 for testing. For the VisAlgae 2023 dataset, the data was partitioned into a training-validation set and a test set at a ratio of 7:3, with the training-validation subset further divided into training and validation sets at an 8:2 ratio. In this evaluation, the proposed model, the baseline RT-DETR-R18, and the strongest competitor YOLOv11m were initialized using their respective weights pre-trained on the EMDS-7 dataset and subsequently fine-tuned on the two new datasets. All models were fine-tuned for 50 epochs under the same training protocol, and all remaining hyperparameter settings were kept consistent with those described in Section 4.2.

As shown in Table 5, the proposed method consistently achieved the best detection performance across both external datasets. Most notably, on the VisAlgae 2023 dataset, our model reached an mAP@0.5 of 91.94%, outperforming the baseline RT-DETR-R18 and YOLOv11m by clear margins. These quantitative results suggest that the performance advantages of the proposed method are maintained across different microscopic image domains.

## 5. Discussion

Cyanobacteria detection in microscopic images remains difficult because the targets vary markedly in scale and morphology and are often embedded in algae-rich aquatic backgrounds. In the same field of view, cyanobacteria may appear as isolated cells, compact colonies, or elongated filaments, while some non-target structures show similar local textures or contours [22,25,45]. These characteristics make both manual inspection and automated detection challenging and place joint demands on feature discrimination, multi-scale representation, and robustness to background interference.

A plausible explanation for the observed results is that SeFaster contributes to reducing background redundancies, HSFPN appears to facilitate cross-scale interactions for differently sized targets, and WIoU helps mitigate the impact of uneven sample quality on localization learning. The ablation results show that each component contributes positively, and that their combination yields the best performance, indicating that backbone refinement, feature fusion, and regression optimization are complementary. One important finding is that the proposed model improves accuracy without increasing model complexity relative to the baseline RT-DETR-R18. This is relevant for practical cyanobacteria monitoring, where throughput and deployment cost matter in addition to accuracy. Compared with the baseline, the proposed model improves mAP while reducing parameters and FLOPs and increasing FPS, suggesting a better balance between detection performance and computational efficiency.

**Figure 7:**
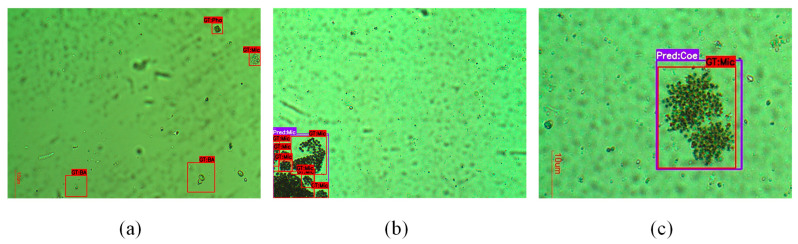
Failure Cases Analysis on representative EMDS-7 test images. Subfigures (**a**–**c**) correspond to image IDs G003-138, G003-293, and G030-036, respectively. Red boxes denote ground-truth annotations, and purple boxes denote predictions from the proposed model.

As reported in Table 3, the proposed method achieved the highest mAP@0.5 across the seven cyanobacteria categories. The improvement was more evident for visually representative and relatively well-sampled categories, such as *Oscillatoria* and *Microcystis*. In contrast, categories with fewer samples, such as *Raphidiopsis* and *Anabaenopsis*, showed less stable differences among models, making their per-class mAP values more sensitive to sample distribution. *Coelosphaerium* remained challenging for most models, indicating that this category is still difficult under the current dataset and detection setting. Despite the overall performance gains, several limitations remain in handling complex real-world samples. To further examine the model errors, representative failure cases from the test set are shown in Figure 7. In Figure 7a, the image contains small targets together with bubbles and suspended debris. Since small circular or oval cyanobacteria may share similar local appearance with these impurities, the model tends to make conservative predictions, resulting in missed detections in low-contrast or noisy regions. Figure 7b illustrates a dense *Microcystis* cluster with severe spatial overlap. Although the model captures the main *Microcystis* region, the prediction does not exactly match the individual ground-truth boxes used in the evaluation. Therefore, some detections may be penalized by the IoU-based metric even when the biologically relevant colony region is broadly localized. This suggests that colonial genera such as *Microcystis* and *Coelosphaerium* remain difficult for box-level object detection, especially when adjacent individuals merge into compact clusters. In Figure 7c, slight defocus weakens fine structural details and contributes to confusion between *Coelosphaerium* and *Microcystis*, which both exhibit colonial and approximately spherical morphology.

Consistent with these qualitative observations, the detection difficulty is also highly dependent on object scale. A supplementary quantitative evaluation, following the standard COCO evaluation metrics [46], is provided in Table S3 of the Supplementary Materials. It is important to note that small targets represent an extreme minority in the dataset, with only 9 instances in total. Therefore, the mAP@0.5:0.95 yielded by all models on small targets lacks statistical significance. For medium and large targets, the proposed model consistently outperforms the baseline and the strongest competitor, YOLOv11m, which clearly demonstrates the overall effectiveness of the proposed method. However, the imbalance among small, medium, and large scales under the current dataset split may be one of the reasons restricting the model from achieving even better performance. Nevertheless, the supplementary evaluation on all 42 original EMDS-7 subcategories further supports the effectiveness of the proposed model. Compared with the best-performing model reported in the original EMDS-7 study [25], the proposed method achieved higher or comparable AP in 28 subcategories, with an average AP improvement of 6%. These results suggest that the proposed framework has good application potential beyond the reorganized seven-category cyanobacteria setting. However, the failure cases also show that improvements in computer vision and AI alone cannot completely eliminate the difficulties caused by real-world water samples. Since EMDS-7 was collected from natural aquatic environments, complex backgrounds, suspended impurities, overlapping colonies, and variations in image quality inevitably increase detection difficulty. Another practical limitation is that not all cells are perfectly flat in field samples, causing recognition to depend on expert experience or training with both good and poor imagery for reliable field sample assessment.

Furthermore, the cross-dataset transfer experiments detailed in Section 4.6 provide supporting evidence regarding the stability and robustness of the model. A common challenge in deep learning for microscopic image analysis is the tendency to achieve optimal performance exclusively on a specific dataset or under a coincidental data split. However, the consistent superiority of our model on the independent Marine-MicroalgaeDetection and VisAlgae 2023 datasets suggests that the observed performance gains are not merely an artifact of the EMDS-7 data division, but rather reflect improved modeling capability and transferability across different microscopic image domains. Nevertheless, the geographic generalizability of the model remains to be further validated. Variations in local climates, water chemistry, and cyanobacterial strain composition may introduce additional domain shifts that were not represented in the evaluated datasets. Therefore, transfer learning and fine-tuning with locally collected samples may facilitate efficient adaptation to new environmental conditions.

Therefore, future work should not only improve the algorithm but also consider the biological and experimental workflow. Expanding and balancing the dataset remains important, especially for underrepresented categories, difficult background cases, non-flat cell presentations, and images of varying quality. In addition, stronger instance separation strategies or detection–segmentation integrated frameworks may help address dense and overlapping targets. Furthermore, extending the current object detection results into standardized biological quantitation remains an important direction for future work. From the sample-processing perspective, biologically informed preprocessing procedures, such as size-based filtering or expert-guided sample screening [11,47], may reduce visual interference before imaging. Overall, this study suggests that microscopy image-based visual detection, when combined with an effective AI model, provides a promising approach for cyanobacteria detection. Future development should further integrate AI methods with biological expertise to improve robustness in practical water-quality monitoring scenarios.

## 6. Conclusions

This study proposed an adaptive multi-scale fusion enhanced RT-DETR framework for cyanobacteria detection in microscopic images. To address the main challenges of this task, including redundant aquatic background, large scale variation, and uneven sample quality, three targeted modifications were introduced into the baseline RT-DETR-R18. Specifically, SeFaster was used to improve backbone feature extraction efficiency, HSFPN was introduced to strengthen semantic-guided multi-scale fusion, and WIoU was adopted to improve localization learning under mixed sample quality. Experiments on the reorganized EMDS-7 dataset showed that the proposed method outperformed the compared YOLO-family and non-YOLO detectors in overall detection performance. Relative to the baseline RT-DETR-R18, the proposed model improved mAP while reducing FLOPs and parameter count and increasing inference speed. The per-class results further showed the highest mAP across the seven cyanobacteria categories, and the ablation results confirmed that all three proposed components contributed positively to the final performance. Overall, the results indicate that adapting Transformer-based detection to the visual characteristics of cyanobacterial microscopic images is an effective direction for improving both accuracy and efficiency. Further work is still needed on class imbalance, difficult categories, and cross-domain validation, but the present framework demonstrates potential as a useful basis for automated cyanobacteria monitoring in microscopic image analysis.

## Figures and Tables

**Table 1:** Class distribution of the reorganized EMDS-7 dataset used in this study.

Class	Abbreviation	Images Number	Instances Number	Representative Example
Oscillatoria (G001)	Osc	41	178	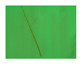
Phormidium (G022)	Pho	276	1216	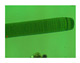
Spirulina (G015)	Spi	18	73	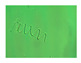
Microcystis (G003)	Mic	307	826	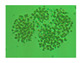
Coelosphaerium (G025)	Coe	77	165	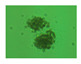
Anabaenopsis (G024)	Ana	22	37	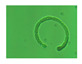
Raphidiopsis (G034)	Rap	9	19	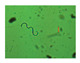
Background Algae (G002, G004, G005, G006, G007, G008, G009, G010, G011, G014, G016, G017, G018, G019, G020, G021, G023, G026, G027, G028, G029, G031, G032, G033, G035, G036, G040, G041)	BA	1279	2225	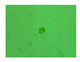 e.g., Chlorella
Zooplankton (G012, G013, G030, G037, G038, G039)	Zoo	336	389	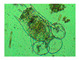 e.g., Brachionus

Note: Background Algae denotes the algae-dominated background category formed by merging non-target algal classes in EMDS-7, whereas Zooplankton denotes non-algal aquatic microorganisms retained as interference categories.

**Table 2:** Comparison of overall detection performance on the reorganized EMDS-7 dataset.

Model	P (%)	R (%)	mAP@0.5 (%)	mAP@0.5:0.95 (%)	FLOPs (G)	Param (M)	FPS
YOLOv8m [34]	83.23	69.67	77.05 [70.47, 81.38]	65.02 [58.84, 69.02]	79.1	25.86	55.06
YOLOv10m [35]	79.98	66.44	73.03 [65.40, 77.33]	60.78 [53.89, 65.02]	64.0	16.49	62.92
YOLOv11m [36]	**83.92**	72.67	77.74 [70.25, 81.47]	65.14 [58.79, 70.23]	68.2	20.06	63.98
YOLOv12s [37]	73.50	71.19	73.47 [66.5, 78.26]	58.90 [53.14, 63.69]	21.5	9.26	**71.27**
YOLOv12m [37]	79.66	70.19	77.51 [70.99, 81.59]	64.84 [58.3, 69.02]	67.8	20.14	45.64
YOLOv13s [38]	76.15	62.39	70.27 [63.45, 74.93]	55.38 [49.16, 61.12]	**21.0**	**9.03**	65.61
YOLO26s [39]	76.50	66.36	68.10 [61.55, 72.22]	57.84 [51.56, 62.01]	22.5	9.9	70.71
YOLO26m [39]	82.65	69.68	73.39 [66.27, 77.83]	62.69 [56.78, 68.13]	74.8	21.79	59.12
RF-DETR-S [41]	82.01	59.87	76.36 [69.34, 80.32]	63.95 [57.67, 68.53]	79.2	33.4	63.06
D-FINE-S [40]	57.97	59.17	72.30 [65.34, 76.56]	59.12 [52.98, 64.98]	24.9	10.18	70.11
RT-DETR-R18 [30]	81.06	70.00	75.85 [69.85, 80.64]	61.20 [56.21, 66.56]	58.3	20.10	66.27
Ours	83.70	**73.67**	**79.05** [72.9, 83.77]	**66.03** [59.99, 70.95]	54.6	16.31	70.85

Note: The best and second-best results are highlighted in bold and underlined, respectively. Values in brackets represent the 95% confidence intervals calculated via bootstrap iterations.

**Table 3:** Per-class AP@0.5 (%) and mAP@0.5 (%) over the seven cyanobacteria classes.

Models	Osc	Pho	Spi	Mic	Coe	Ana	Rap	mAP@0.5 of Cyanobacteria	BA	Zoo
YOLOv8m [34]	62.43	**78.34**	70.3	63.63	57.45	**80.08**	99.5	73.1	84.17	**97.56**
YOLOv10m [35]	59.1	67.30	74.31	56.68	56.1	72.59	99.5	69.37	79.74	91.96
YOLOv11m [36]	65.57	75.31	67.08	67.51	**64.14**	77.5	99.5	73.8	86.49	96.58
YOLOv12s [37]	59.95	77.02	53.25	62.64	56.02	75.75	99.5	69.16	83.02	94.05
YOLOv12m [37]	56.04	78.76	73.59	68.54	61.33	77.20	99.5	73.57	86.34	96.29
YOLOv13s [38]	44.85	69.71	52.20	64.16	54.54	74.94	99.5	65.7	80.29	92.21
YOLO26s [39]	40.86	66.4	61.21	53.73	50.99	72.78	99.5	63.64	74.99	92.41
YOLO26m [39]	65.29	69.64	67.20	57.03	56.71	71.50	99.5	69.55	77.82	95.82
RF-DETR-S [41]	65.01	74.4	72.65	66.82	53.29	77.28	99.5	72.71	84.43	93.88
D-FINE-S [40]	61.73	75.71	76.09	63.94	32.58	62.11	99.5	67.38	82.72	96.28
RT-DETR-R18 [30]	58.89	72.98	**82.82**	68.43	48.95	75.41	99.5	72.43	83.62	91.96
Ours	**71.58**	75.04	80.47	**69.87**	54.99	75.73	99.5	**75.31**	**87.35**	96.89

Note: The best and second-best results are highlighted in bold and underlined, respectively.

**Table 4:** Ablation results of data augmentation and different components on the re-organized EMDS-7 dataset.

No.	Data Aug	SeFaster	HSFPN	WIoU	FLOPs (G)	Param (M)	P (%)	R (%)	mAP@0.5 (%)	mAP@0.5:0.95 (%)
1	-	-	-	-	58.3	20.10	59.39	64.58	61.38	50.78
2	✓	-	-	-	58.3	20.10	81.06	70.00	75.85	61.20
3	✓	✓	-	-	**50.9**	17.10	81.75	71.08	76.91	62.48
4	✓	-	✓	-	61.9	19.32	81.66	70.84	76.71	63.91
5	✓	-	-	✓	58.3	20.10	83.40	72.58	76.95	62.67
6	✓	✓	✓	-	54.6	**16.31**	83.64	72.95	77.73	64.80
7	✓	✓	-	✓	**50.9**	17.10	83.28	72.93	77.61	63.36
8	✓	-	✓	✓	61.9	19.32	82.43	71.51	77.71	64.13
9	✓	✓	✓	✓	54.6	**16.31**	**83.70**	**73.67**	**79.05**	**66.03**

Note: “✓” indicates that the corresponding data augmentation strategy or component was used, while “-” indicates that it was not used. The best and second-best results are highlighted in bold and underlined, respectively.

**Table 5:** Comparison of cross-dataset transfer performance on the Marine-MicroalgaeDetection and VisAlgae 2023 datasets.

Models	Marine-MicroalgaeDetection (%)				VisAlgae 2023 (%)			
	P	R	mAP@0.5	mAP@0.5:0.95	P	R	mAP@0.5	mAP@0.5:0.95
YOLOv11m [36]	71.20	77.27	77.69	51.18	76.70	76.01	76.99	56.32
RT-DETR-R18 [30]	67.55	79.08	75.82	51.96	84.21	80.10	82.29	60.18
Ours	**79.13**	**83.41**	**82.01**	**56.04**	**89.56**	**85.97**	**91.94**	**66.56**

Note: The best and second-best results are highlighted in bold and underlined, respectively.

## Data Availability

The publicly available datasets analyzed in this study can be accessed through the following sources: the EMDS-7 dataset is available at https://figshare.com/articles/dataset/EMDS-7_DataSet/16869571 (accessed on 5 June 2026); the VisAlgae 2023 dataset can be found at https://github.com/juntaoJianggavin/Visalgae2023 (accessed on 5 June 2026); and the Marine-MicroalgaeDetection dataset is provided at https://tianchi.aliyun.com/competition/entrance/532036/introduction (accessed on 5 June 2026). The code that supports the findings of this study is publicly available at https://github.com/qfxlsbbx2025/An-Enhanced-RT-DETR-for-Efficient-Cyanobacteria-Detection (accessed on 5 June 2026).

## Supplementary Materials

The following supporting information can be downloaded at: https://www.mdpi.com/article/10.3390/biology15120970/s1, Figure S1. The blue bars represent the Faster RCNN benchmark reported in the original EMDS-7 study, and the orange bars represent the proposed model. Table S1. Quantitative comparison of average precision (AP) on the 42 EMDS-7 subcategories. Table S2. Detailed distribution of images and instances across train, validation, and test splits. Table S3. Quantitative performance comparison by object size (mAP@0.5:0.95, %).

## Abbreviations

The following abbreviations are used in this manuscript:

RT-DETR	Real-Time Detection Transformer
AIFI	Attention-based Intra-scale Feature Interaction module
CCFM	CNN-based Cross-scale Feature Fusion module
SeFaster	squeeze and excitation faster feature extraction module
HSFPN	high-level screening-feature fusion pyramid network
WIoU	Wise-IoU
EMDS-7	Environmental Microorganism Image Dataset Seventh Version
P	Precision
R	Recall
mAP	mean Average Precision
