# Supplementary material for: Adaptive Multi-Scale Fusion Enhanced RT-DETR for Efficient Cyanobacteria Detection in Microscopic Images
Source: Biology (Basel). 2026 Jun 20;15(12):970. doi: 10.3390/biology15120970 (PMC13295691; doi:10.3390/biology15120970)
Supplement: Supplementary file 1 [file biology-15-00970-s001.zip › biology-4337185-supplementary.pdf]

Supporting Information

Adaptive Multi-Scale Fusion Enhanced RT-DETR for Efficient Cyanobacteria Detection in Microscopic Images

Jianxing Li [1,2], Shizhi Zheng [1,2], Yu Chen [3,*], and Kan Luo [1,2,*]

[1] School of Electrical and Information Engineering, Fujian University of Technology, Fuzhou, China; lijx@fjut.edu.cn (J.L.); 2241905011@smail.fjut.edu.cn (S.Z.); luokan@fjut.edu.cn (K.L.)

[2] Fuzhou Industrial Integration Automation Technology Innovation Center, Fuzhou, China

[3] State Key Laboratory of Digital Medical Engineering, School of Instrument Science and Engineering, Southeast University, Nanjing, China; 230268561@seu.edu.cn (Y.C.)

* Correspondence: 230268561@seu.edu.cn (Y.C.); luokan@fjut.edu.cn (K.L.)

S1 Subcategory-level evaluation on the EMDS-7 dataset.

To further validate the detection performance of the proposed model, we conducted a fine-grained evaluation on all 42 subcategories of the EMDS-7 dataset. The Average Precision (AP) is used as the primary evaluation metric, which is calculated as the area under the Precision-Recall (P-R) curve, as shown in Equation (S1):

$$AP = \int_0^1 P(R)dR \tag{S1}$$

where P denotes Precision and R denotes Recall.

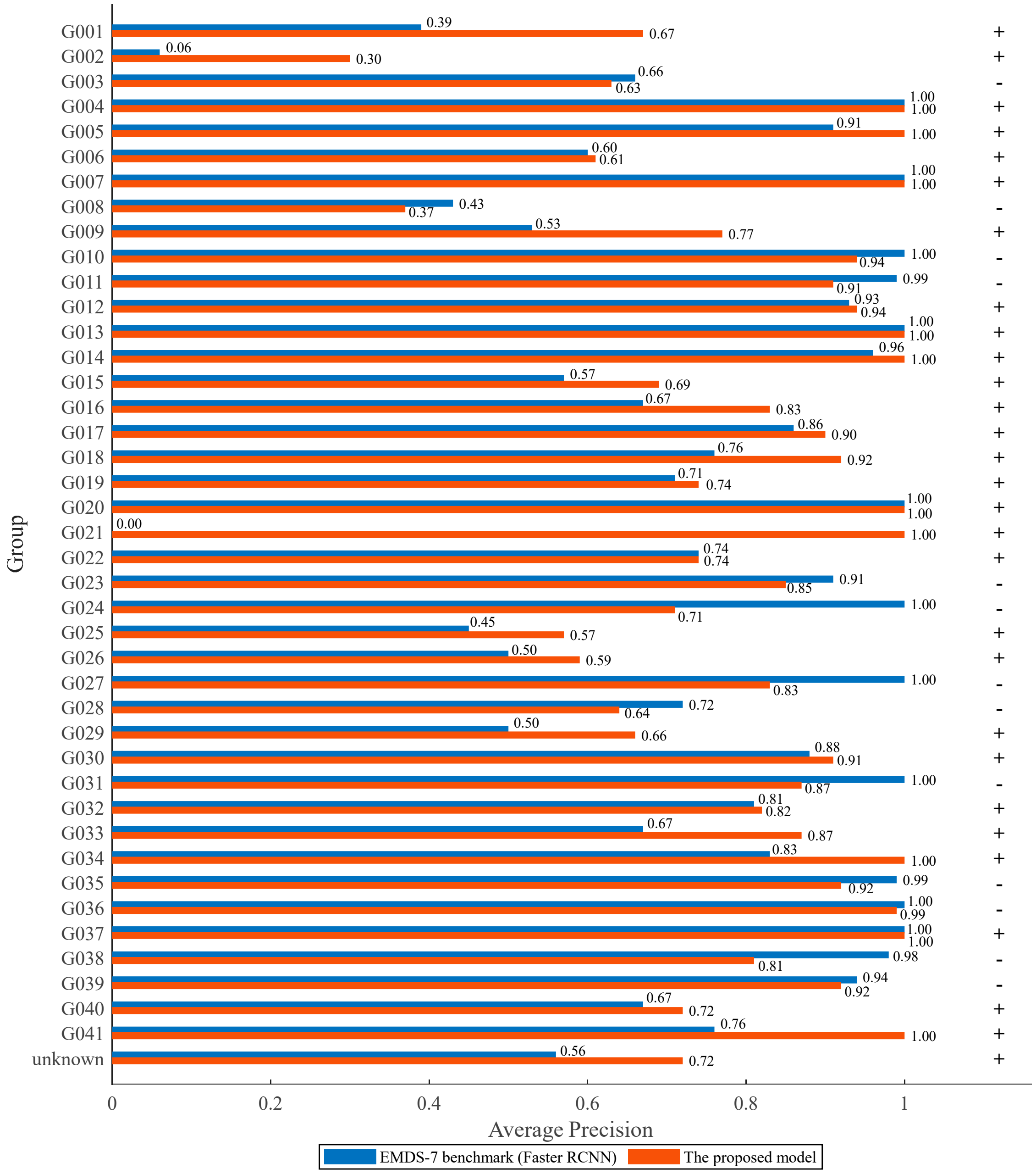

Figure S1. The blue bars represent the Faster RCNN benchmark reported in the original EMDS-7 study, and the orange bars represent the proposed model. A "+" indicates that the proposed model achieved equal or higher AP than the Faster RCNN benchmark for that subcategory, whereas a "−" indicates lower AP. Overall, the proposed model achieved higher or comparable AP in 28 subcategories.

Table S1. Quantitative comparison of average precision (AP) on the 42 EMDS-7 subcategories.

	**Yolo v3***	**Yolo v4***	**SSD***	**RetinaNet***	**Faster RCNN***	**Proposed model**
G001	19	0	2	4	<u>39</u>	**67**
G002	0	0	0	0	<u>6</u>	**30**
G003	57	45	46	45	**66**	<u>63</u>
G004	**100**	95	84	94	**100**	<u>100</u>
G005	89	72	91	91	<u>91</u>	**100**
G006	40	20	<u>60</u>	<u>60</u>	<u>60</u>	**61**
G007	50	**100**	**100**	**100**	**100**	<u>100</u>
G008	**45**	28	29	35	<u>43</u>	37
G009	50	43	46	47	<u>53</u>	**77**
G010	**100**	86	**100**	**100**	**100**	<u>94</u>
G011	97	83	80	<u>98</u>	**99**	91
G012	76	49	66	66	<u>93</u>	**94**
G013	25	**100**	0	<u>50</u>	**100**	**100**
G014	94	82	<u>96</u>	<u>96</u>	<u>96</u>	**100**
G015	42	21	29	40	<u>57</u>	**69**
G016	52	45	57	62	<u>67</u>	**83**
G017	72	62	65	63	<u>86</u>	**90**
G018	73	74	<u>80</u>	75	76	**92**
G019	<u>71</u>	38	69	61	<u>71</u>	**74**
G020	83	<u>86</u>	71	<u>86</u>	**100**	**100**
G021	0	0	0	0	0	**100**
G022	64	49	43	48	**74**	<u>74</u>
G023	**91**	43	91	82	**91**	<u>85</u>
G024	<u>94</u>	40	80	**100**	**100**	71
G025	24	2	44	19	<u>45</u>	**57**
G026	42	0	<u>50</u>	<u>50</u>	<u>50</u>	**59**
G027	67	0	50	67	**100**	<u>83</u>
G028	56	31	31	34	**72**	<u>64</u>
G029	<u>50</u>	0	12	25	<u>50</u>	**66**
G030	71	22	87	50	<u>88</u>	**91**
G031	63	83	<u>98</u>	83	**100**	87
G032	70	28	78	75	<u>81</u>	**82**
G033	<u>67</u>	<u>67</u>	<u>67</u>	50	<u>67</u>	**87**
G034	0	0	50	0	<u>83</u>	**100**
G035	71	16	91	77	**99**	<u>92</u>
G036	93	80	93	73	**100**	<u>99</u>
G037	50	0	50	50	**100**	<u>100</u>
G038	52	23	69	68	**98**	<u>81</u>
G039	82	40	72	85	**94**	<u>92</u>
G040	0	0	17	0	<u>67</u>	**72**
G041	60	0	60	0	<u>76</u>	**100**
unknown	<u>58</u>	38	24	36	56	**72**
Mean AP	59	40	58	56	<u>76</u>	**82**

Note: * indicates results reported in the original EMDS-7 study. The best and second-best results are highlighted in bold and underlined, respectively. To maintain consistency with the Faster RCNN benchmark reported in the literature, the AP values were rounded to two decimal places and are presented here as integer percentages.

As shown in Figure S1 and Table S1, the proposed model achieved the highest mean AP of 82%, outperforming the five benchmark detectors reported in the original EMDS-7 study [25]. Compared

with the best benchmark model (Faster RCNN) in reported study, the proposed model improved the mean AP from 76% to 82%, corresponding to an absolute gain of 6%. It also outperformed YOLOv3, YOLOv4, SSD, and RetinaNet, which achieved mean AP values of 59 %, 40%, 58%, and 56%, respectively.

In the subcategory-wise comparison with Faster RCNN, the proposed model achieved higher or comparable AP in 28 subcategories. Clear improvements were observed in several difficult categories, such as G001, G002, G009, G021, G033, and G041. These results demonstrate that the proposed model provides a clear improvement over existing benchmark detectors, particularly for subcategories with relatively low baseline performance.

S2 Detailed dataset division and instance distribution

To provide a comprehensive overview of the dataset composition, Table S2 details the exact distribution of images and bounding box instances across the training, validation, and test splits.

Table S2. Detailed distribution of images and instances across train, validation, and test splits.

	**Original Train**		**Validation**		**Test**	
**Class**	**Images**	**Instances**	**Images**	**Instances**	**Images**	**Instances**
Oscillatoria	25	111	8	30	8	37
Phormidium	166	755	55	224	55	237
Spirulina	10	50	4	10	4	13
Microcystis	185	488	61	161	61	177
Coelosphaerium	47	90	15	38	15	37
Anabaenopsis	14	20	4	9	4	8
Raphidiopsis	5	14	2	2	2	3
Background Algae	765	1341	257	423	257	461
Zooplankton	200	235	68	76	68	78
Total	1417	3104	474	973	474	1051

S3 Quantitative evaluation across different object scales

To further investigate the model performance under varying target dimensions, Table S3 presents a quantitative comparison based on object size (Small, Medium, and Large). As detailed in the Discussion section of the main manuscript, the proposed model consistently outperforms the baselines on medium and large targets.

Table S3. Quantitative performance comparison by object size (mAP@0.5:0.95, %).

**Object Size**	**Small**	**Medium**	**Large**
**Number of Instances (Original Train/Val/Test)**	6/2/1	636/179/218	2462/792/832
YOLOv8m[34]	0	35.6	66.1
YOLOv10m[35]	0	35.7	62.0
YOLOv11m[36]	0	35.9	<u>66.6</u>
YOLOv12s[37]	0	30.0	62.9
YOLOv12m[37]	0	33.2	66.5
YOLOv13s[38]	0	27.0	58.5
YOLO26s[39]	0	26.8	60.9
YOLO26m[39]	0	34.6	65.4
RF-DETR-S[41]	0	33.5	66.3
D-FINE-S[40]	0	28.8	60.8
RT-DETR-R18[30]	0	<u>40.6</u>	63.1
Ours	0	**41.5**	**66.9**

Note: Object scales are defined according to the standard MS COCO evaluation metrics: Small (area $< 32 \times 32$ pixels), Medium ($32 \times 32 \leq$ area $\leq 96 \times 96$ pixels), and Large (area $> 96 \times 96$ pixels) [46]. The best and second-best results are highlighted in bold and underlined, respectively.
